# Maxillary Sinus Natural Killer/T-Cell Extranodal Lymphoma Mimicking a Dentoalveolar Abscess

**DOI:** 10.1155/2019/6701783

**Published:** 2019-02-27

**Authors:** Erika Antonia dos Anjos Ramos, Luciana Munhoz, Basílio Almeida Milani, Tomás Zecchini Barrese, Stephanie Kenig Viveiros, Emiko Saito Arita

**Affiliations:** ^1^Department of Stomatology, School of Dentistry, University of São Paulo, 2227 Lineu Prestes Avenue, 05508-000 São Paulo, SP, Brazil; ^2^Department of Maxillofacial Surgery, Hospital Municipal do Campo Limpo, 1661 Estrada da Itapecerica, 05835-005 São Paulo, Brazil; ^3^Department of Pathological Sciences, School of Medicine, Santa Casa de Sao Paulo School of Medical Sciences, 61 Dr. Cesário Motta Junior Street, 01221-020 São Paulo, SP, Brazil

## Abstract

Extranodal natural killer/T-cell lymphoma (ENKL) is an aggressive and infrequent malignant neoplasm. Early sinonasal ENKL clinical symptomatology is often not specific which can mimic several clinical odontogenic processes such as dentoalveolar abscesses. A 41-year-old female was referred to our institution due to facial pain with skin rash, fever, and intraoral swelling in the left side of the maxillary region. Computed tomography (CT) revealed a soft tissue hypodense area in the left side with bone discontinuity in anterior and lateral maxillary sinuses. Initial laboratorial tests showed no alterations on hemogram, coagulation profile, and immune phenotype (CD3^+^/CD4^+^). However, the lesion progressed fastly showing an evident growth, so it was decided that an intraoral biopsy should be performed. The diagnosis was sinonasal ENKLs. This case is an example that the rare T-cell lymphoma can mimic an odontogenic lesion, thus puzzling the clinician. Fortunately, the rapid growth of the lesion prompted the correct diagnosis and early treatment.

## 1. Introduction

Extranodal natural killer/T-cell lymphoma (ENKL) is an infrequent malignant neoplasm [1] that arises primarily from the natural killer cell [2] and accounts for 15% of all non-Hodgkin lymphomas located in the nasal fossae and maxillary sinuses [3]. The sinonasal ENKL location is unusual [4]. Formerly, the lesion was not recognized as a neoplastic process, and it was named “malignant centrofacial granuloma,” [4] “lethal midline granuloma,” or angiocentric lymphoma, due to its necrotizing and invasive evolution features [2].

Sinonasal ENKL presents as a highly aggressive neoplasm [2], which is characterized by the progressive destruction of the midline facial and/or nasal structures [3, 5] and eventually also may affect the orbit by contiguity [3]. Early sinonasal ENKL clinical symptomatology is often not specific and may include headache, facial pain, nasal congestion, and fever [1]. Although most of the sinonasal ENKL patients exhibit locoregional disease, systemic involvement may be present [5].

Due to the anatomical proximity with oral cavity and paranasal sinuses, ENKL can affect intraoral elements, which can mimic several clinical odontogenic origin entities, including inflammatory and infectious diseases, such as dentoalveolar abscesses [6].

Accordingly, the objective of this report is to describe the main clinical, radiographic, and histologic characteristics of a sinonasal ENKL case in the maxillary sinuses, first diagnosed as an odontogenic abscess due to its early clinical features. Furthermore, the aggressive lesion development is also illustrated in tomographic examinations performed in different short time interval moments.

## 2. Case Report

A 41-year-old Caucasian woman noticed left cheek swelling and pain in corresponding upper premolars and molars and attended to a consultation in the Oral and Maxillofacial service at Hospital Municipal do Campo Limpo (São Paulo, Brazil). Her medical history revealed a previously ischemic cerebrovascular accident and continuous use of anticoagulants but no other past disease.

**Figure 1:**
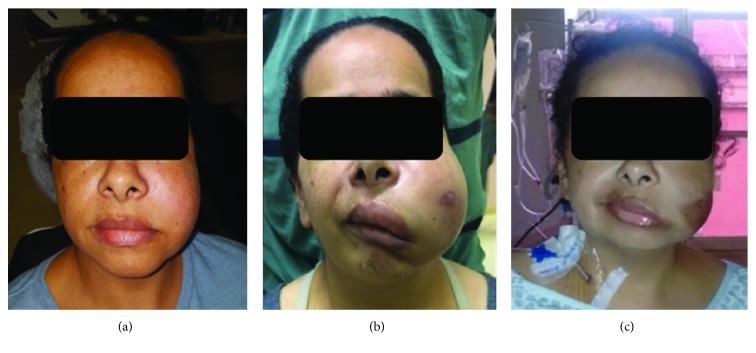
From left to right: (a) exhibits patient facial aspect at the first consultation, note the slight bulging in the left side of the face; (b) demonstrates the lesion fast progress by showing the evident lesion growth in 3 weeks; (c) shows lesion shrink after 4 weeks of chemotherapy.

Extraoral examination showed no lymphadenopathy or paresthesia but an evident swelling in the left cheek (Figure 1(a)) with a hardened consistency. The patient mentioned she noticed the symptoms for a period of time higher than a week. Intraorally, she showed severe periodontal disease and pain during vertical/horizontal percussion tests. Pulp vitality could not be determined at this point, due to the severity of pain presented during teeth percussion tests. Buccoalveolar sulcus bulging and a floating tumefaction in the left upper molar root region were observed. Although the first diagnostic hypothesis was dental abscess, due to the unusual duration of the tumefaction, the patient was then referred to imaging examinations (multislice computed tomography—MCT). However, due to the presence of an intraoral floating tumefaction and dental origin symptomatology, drainage was performed, and antibiotic treatment was prescribed.

Despite of accomplishing imaging examinations as requested in the first consultation, the patient only came back to the Oral and Maxillofacial service after three weeks, when she felt paresthesia and severe pain in her left face.  Figure 1(b) exhibits the patient left face swelling aspect at this moment. Due to the increase in painful symptomatology and evident speedy lesion development, the patient was immediately hospitalized, and a new CT examination was performed, as well as routine and biochemical blood test examinations. Intraoral floating was now absented.

No alterations were verified in the patient hemogram, coagulation tests, and immune phenotype (CD3^+^/CD4^+^) examinations, as well as blood tests for hormones, especially the parathyroid hormone.

### 2.1. Imaging Evaluations

**Figure 2:**
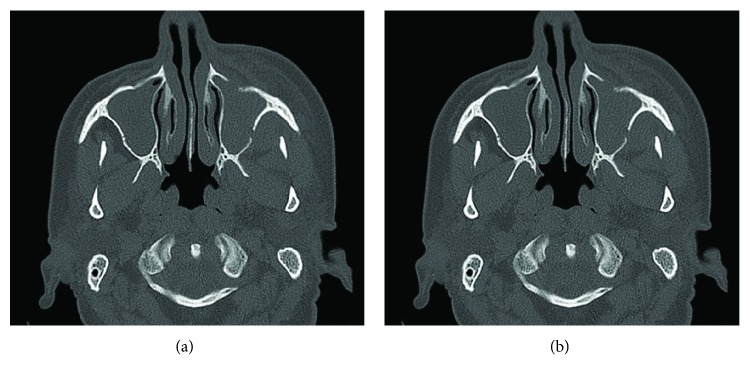
First consultation examination. (a) The axial MCT scan shows a soft-tissue mass anterior to the maxillary sinus, left side, and sinus anterior and lateral wall bone erosion. (b) Coronal MCT scan exhibits nasal cavity involvement by soft-tissue mass, as well as sinus lateral wall bone destruction.

Noncontrast-enhanced, high resolution MCT with 16 slices (Toshiba Activion, Medical Systems Corporation, Japan) was used for MCT imaging. Acquisition imaging parameters were 0.5 mm slice thickness, 1.0 mm *Spacing* Between Slices, 250 mm field of view, 120 kV peak, and 250 mA. The first consultation MCT axial slices revealed a soft tissue hypodense area in the left side, comprising the maxillary sinus area and bone discontinuity in the anterior and lateral maxillary sinus wall (Figure 2(a)) orbital cavity floor. Frontal slices showed discrete nasal cavity involvement (Figure 2(b)).

The second consultation MCT axial slices revealed that the soft tissue hypodense area in the left side increased in size, with greater nasal cavity involvement and bone destruction than Figure 2.

At this point, the main diagnostic hypothesis was osteosarcoma, sinonasal diffuse large B-cell lymphoma, parathyroid brown tumor, and extranodal malignant lymphoma, particularly maxillary ENKL. The hypothesis of dentoalveolar abscess was disregarded: floating tumefaction might be coincidentally previously present due to the periodontal disease severe status.

### 2.2. Histopathological Results/Histopathology

A biopsy was taken from the lesion (oral cavity) and sent to the hospital's pathology department. The histopathological examination with hematoxylin and eosin (H&E) showed a diffuse proliferation of small to medium-sized atypical lymphocytes with areas showing an angiocentric pattern effacing the normal architecture of the oral mucosa. Mild epithelium tropism and espongiosis were observed (Figures 4(a) and 4(b)). Focal areas of ulceration and necrosis were also seen. Trial immunohistochemical and in situ hybridization reactions were made (Figures 4(c)–4(i)). The findings were consistent with ENKL. As part of staging, a bone marrow biopsy was done, and no abnormalities were found.

### 2.3. Treatment

Before the diagnostic definition, the patient was treated with one cycle of CHOP (cyclophosphamide, doxorubicin, vincristine, and prednisolone). Once the diagnosis was established, the AspaMetDex (L-asparaginase, methotrexate, and dexamethasone) regimen was started, but no response was observed after two cycles of chemotherapy; on the contrary, the lesion became larger, and the acute inflammatory signs turned more evident. Facing this, the DICE (dexamethasone, isofosfamide, cisplatin, and etoposide) regimen and local radiotherapy (30 Gy) were started. After two cycles, the lesion presented a significant reduction, but her general condition deteriorated and was associated with mucositis and persistent neutropenia. Before even starting another cycle, five months after the diagnosis, the patient passed away.

## 3. Discussion

Although malignant lymphomas are also considered opportunistic neoplasms as they usually affect immunocompromised patients [7], in the present case, there were no history, blood laboratory results, or signs that suggested compromised immunity, as previously described in the literature [1].

Sinonasal ENKL is an aggressive and speedy evolution lesion [1]. Our case confirms this, demonstrating the fast disease progress clinically observed in three weeks and at MCT. Furthermore, due to the anatomical proximity with the oral cavity, sinonasal ENKL may compromise intraoral structures and, eventually, in the presence of a concomitant odontogenic disease, mimic infectious diseases [6], such as dentoalveolar abscesses, as specified in the case described. Lymphomas and infectious diseases may present similar MCT images, mainly during the early disease stage, such as the slight bone destruction [6]. Furthermore, clinical symptoms such as mobile teeth, gingival enlargement, oral masses, and swelling next to the oral cavity as chief complaints may lead clinicians to misdiagnose lymphomas as dentoalveolar abscesses [8] in an initial consultation which may delay the final diagnosis.

**Figure 3:**
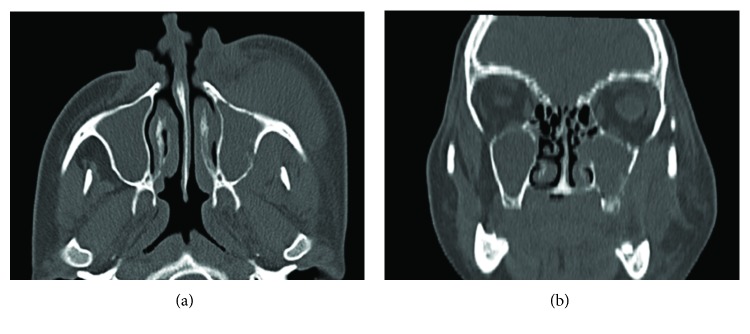
MCT examination 3 weeks after the first consultation. (a) Axial MCT scan, note the prominent increasing of extensive soft tissue. In (b), the nasal cavity involvement is even more evident when compared to the first consultation imaging examination.

**Figure 4:**
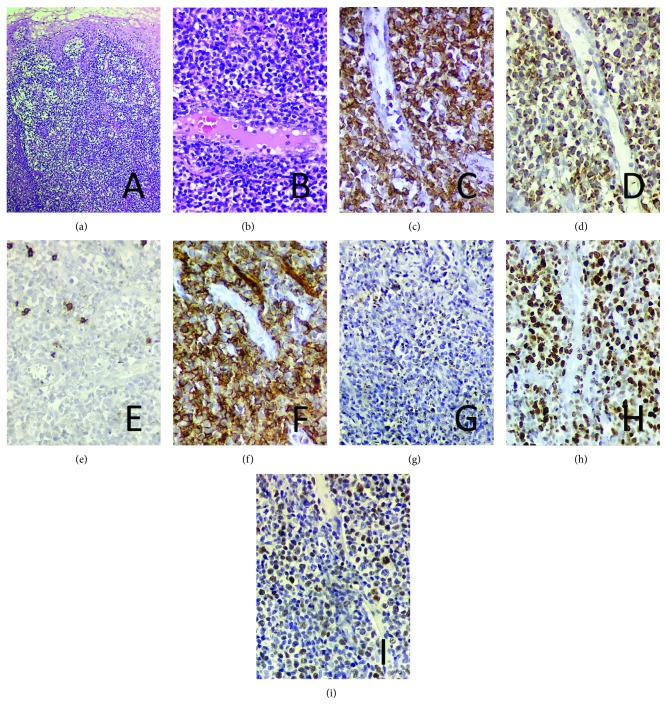
Histopathological features: in (a), the histologic section from the lesion biopsy sample shows a diffuse and permeative infiltrate of small to medium-sized atypical lymphocytes (H&E, ×100); in (b), an evident angiocentric and angiodestructive growth pattern is represented (H&E, ×400). Immunohistochemical and in situ hybridization reactions: in (c), the neoplastic cells show immunopositivity for CD45 (×400); in (d), immunopositivity (partial/weak) for CD3 (×400); in (e), immunonegativity for CD20 (×400); in (f), immunopositivity for CD56 (×400); in (g), immunopositivity (cytoplasmic granules) for TIA1 (×400); in (h), Ki67 index (×400); and in (i), positive nuclear labeling for in situ hybridization for EBV (×400).

Imaging features in MCT often shows infiltrative or permeative bony discontinuity, with different destruction degrees [9]; in our case, evident destruction of sinonasal walls was also observed. The presence of the partially preserved sinus wall for the most extensive tumor involvement, as noticed in Figure 3, is also a usual characteristic of sinonasal lymphomas [9], although sinus wall bone erosion can be the result of other aggressive neoplasm [10]. Extensive soft-tissue mass can be observed in sinonasal ENKLs [10] particularly with lesion progression.

The differential diagnosis should include malignant maxillary tumors, such as sinonanasal undifferentiated and nasopharyngeal carcinoma [1], or other midline ulcerative destructive lesions [3], squamous cell carcinoma [9, 10], oral manifestations of Wegner's granulomatosis [1, 4, 10], primary orbit tumors [3], malignant melanoma, adenoid cystic carcinoma [10], and sinonasal diffuse large B-cell lymphomas, which have similar characteristics when compared to sinonasal ENKLs [2]. In the present case, the leading diagnosis hypothesis after imaging examination evaluation was sinonasal ENKL mainly due to the bony wall destruction noticed and concomitant right and left maxillary sinus opacification, resembling to another case previously described, in which sinonasal ENKL has also mimicked an odontogenic origin infection, delaying the final diagnosis [6].

Outcomes for sinonasal malignancies, enclosing ENKL, have improved recently [2]; nonetheless, prognosis is still relatively poor [2, 4], particularly to patients who have systemic disease [5, 6], and even worse in the case of recurrence [5]. Early precise disease diagnosis for any maxillofacial malignant lymphoma is crucial to achieve better treatment results [1, 11, 12].

Overall, paranasal sinus malignant lymphomas usually present nonspecific early clinical symptomatology [2, 12]. The dental professional can play a valuable role in the diagnosis of patients with sinonasal malignancies which warrants immediate referral to an appropriate specialist when the combination of patient clinical signs arouses suspicion, mainly due to the fact that the mucosa of paranasal sinuses is not accessible as the oral mucosa for further inspection [12].

Notwithstanding, considering the aforementioned nonspecific signals and symptoms, the final diagnosis of the sinonasal ENKLs is achieved by histological and immunohistochemical evaluations [1, 6]. The histological features of ENKLs are usually the same no matter the site affected. The lesions tend to have a diffuse and permeative infiltration of atypical lymphocytes that can be small, medium-sized, large, or anaplastic. The neoplastic cells can be accompanied by a mixture of inflammatory cells. An angiocentric and angiodestructive growth pattern is often present. Besides, this entity frequently shows extensive necrotic areas and ulceration, and multiple biopsies may be required until representative material is acquired. Fortunately, and due to the dimensions of the tumor described here, one single biopsy was enough to the diagnosis. Typically, the immunophenotype of ENKT is CD45 positive, surface CD3 negative, CD2 positive, CD5 negative, and, although not specific, CD56 positive. Cytotoxic molecules such as granzyme B, TIA1, and perforin are positive. The diagnosis virtually requires the presence of EBV, and the most reliable way to demonstrate it is the *in situ* hybridization for EBER, since immunostaining for LMP1 is frequently negative [13].

Treatment for ENKL is mainly dependent on the disease stage at the diagnosis and, although controversial [13], the histologic subtype [2] and is not as yet well codified [4]. Radiotherapy and chemotherapy may be applied [4], but it has often unsatisfactory results [5, 6]. Unfortunately, in our case, the patient have not had a satisfactory response by the moment this manuscript was written.

In conclusion, sinonasal ENKL is an infrequent aggressive disease that can resemble to odontogenic origin diseases, which can delay the diagnosis. Imaging examinations are essential to guide diagnostic hypotheses; however, histological and immunohistochemical examinations are essential in the diagnosis process. The speedy neoplasm progress of this malignant disease was illustrated in this report by the CT examinations performed in distinct moments, with short time interval.
